# Vehicle Speed and Length Estimation Errors Using the Intelligent Transportation System with a Set of Anisotropic Magneto-Resistive (AMR) Sensors

**DOI:** 10.3390/s19235234

**Published:** 2019-11-28

**Authors:** Vytautas Markevicius, Dangirutis Navikas, Adam Idzkowski, Donatas Miklusis, Darius Andriukaitis, Algimantas Valinevicius, Mindaugas Zilys, Mindaugas Cepenas, Wojciech Walendziuk

**Affiliations:** 1 Department of Electronics Engineering, Kaunas University of Technology, Studentu St. 50–439, LT-51368 Kaunas, Lithuania; vytautas.markevicius@ktu.lt (V.M.); dangirutis.navikas@ktu.lt (D.N.); a.idzkowski@pb.edu.pl (A.I.); donatas.miklusis@ktu.edu (D.M.); algimantas.valinevicius@ktu.lt (A.V.); mindaugas.zilys@ktu.lt (M.Z.); mindaugas.cepenas@ktu.lt (M.C.); 2 Faculty of Electrical Engineering, Bialystok University of Technology; Wiejska St. 45D, PL-15351 Bialystok, Poland; w.walendziuk@pb.edu.pl

**Keywords:** magnetic field, AMR sensors, piezoelectric PVDF sensors, vehicle speed detection, car length estimation, signal differentiation, Mean Absolute Error

## Abstract

Seeking an effective method for estimating the speed and length of a car is still a challenge for engineers and scientists who work on intelligent transportation systems. This paper focuses on a self-developed system equipped with four anisotropic magneto-resistive (AMR) sensors which are placed on a road lane. The piezoelectric polyvinylidene fluoride (PVDF) sensors are also mounted and used as a reference device. The methods applied in the research are well-known: the fixed threshold-based method and the adaptive two-extreme-peak detection method. However, the improved accuracy of estimating the length by using one of the methods, which is based on computing the difference quotient of a time-discrete signal (representing the changes in the magnitude of the magnetic field of the Earth), is observed. The obtained results, i.e., the speed and length of a vehicle, are presented for various values of the increment Δ*n* used in numerical differentiation of magnetic field magnitude data. The results were achieved in real traffic conditions after analyzing a data set *M* = 290 of vehicle signatures. The accuracy was evaluated by calculating *MAE* (Mean Absolute Error), *RMSE* (Root Mean Squared Error) for different classes of vehicles. The *MAE* is within the range of 0.52 m–1.18 m when using the appropriate calibration factor. The results are dependent on the distance between sensors, the speed of vehicle and the signal processing method applied.

## 1. Introduction

In recent years many intelligent traffic systems have been created using data acquired from magnetic field sensors [1,2], which exploit the magnetoresistance (MR) effect [2] or the magnetoimpedance (MI) effect [3,4], inductive loops [5] or piezoelectric PVDF sensors [6] installed on a road lane.

By processing the measured data, not only the number of passing vehicles can be counted but also their speed, length and even weight can be estimated [7,8].

In previous works [9,10,11], the authors focused on developing a stationary system which was installed on a public road for measuring individual lengths and speeds and identification of vehicles. It was a built-in system operating in normal traffic conditions, which calculated parameters in real-time. Two anisotropic magneto-resistive sensors (AMR) were placed on the road lane at a distance of 0.3 m [10]. The main aim of the research was focused on the signal processing, the metrological analysis of measurement results as well as testing new algorithms aimed at reducing the calculation time of speed and length of the passing vehicles. This calculated time was shorter than 60 ms for each analyzed vehicle and the accuracy of estimating its speed and length was within the range of ±3 km/h.

A comparative analysis of the results of the determined individual speeds of vehicles traveling in road traffic and the times of executing programs based on selected calculation methods was carried out in previously published articles [9,10,11] as well. To determine the speed of vehicles, the method of detecting the delay value of two signals, with respect to each other, of different computational complexity was used. The results of a comparative analysis of many algorithms for determining the speed of vehicles were developed, not only for signals with a large sample size but also for small sample size sections [10]. Linear interpolation was proposed to be applied in order to increase the resolution of the calculated speed results. On the basis of the assessment of the relative speed results average errors (related to the values read from the radar), favorable calculation methods were selected (ensuring satisfactory accuracy with a small number of samples of the processed signal).

In another work [11], the comparative measures *RMSE* (Root Mean Squared Error) and *NRMSE* (Normalized Root Mean Squared Error) of a large set of speed results of individual vehicles obtained by a selected calculation method and a reference method (a 60 fps video camera) were developed. Four methods for detecting the delay value of two signals in relation to each other were implemented. A signal processing algorithm was developed and selected speed estimation methods were compared. The influence of changing the window width (i.e., number of samples) of the filter averaging the signals on the current basis on the size of estimated speed errors at different set threshold values of signals was investigated. The above mentioned results were obtained for two-sensor systems and it was assumed that a vehicle was passing over the sensors at a constant speed, nor accelerating or slowing down or performing steering maneuvers.

**Figure 1:**
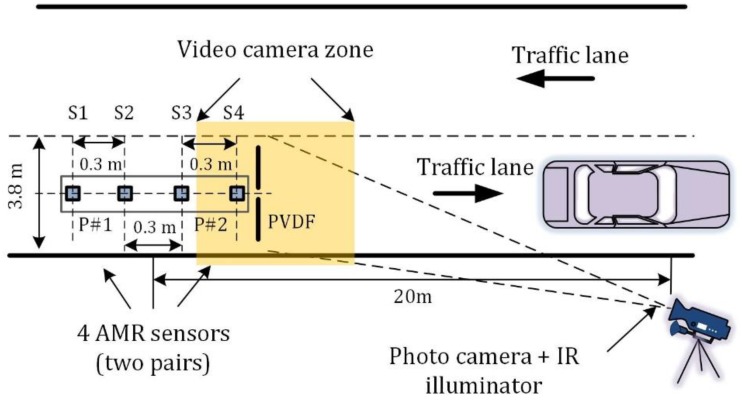
Deployment of traffic monitoring system components on the road (note: used scale does not keep ratio between distance in the figure and the corresponding distance on the sensors placed on the road lane, IR – infrared).

Currently, the authors propose a new solution with two-sensor pairs (Figure 1). The system assures acquiring data with a minimum distance between sensors and is placed on a road lane. Some variants of computations enable more accurate determination of speed and length of a traveling vehicle.

The goal of the research, presented in this paper, is to apply and verify a modified method for speed and length estimation which is based on a fixed or an adaptive thresholding of a signal. It relies on calculating the difference quotient of a time-discrete signal. The initial task was to set up an optimal value of the increment Δ*n* used in numeric differentiation of magnetic field magnitude data. Another goal is to show the differences in speed and length estimation errors which depend on the geometrical parameters of vehicles.

## 2. The Design of a Traffic Monitoring System and Sources of Speed Estimation Error

**Figure 2:**
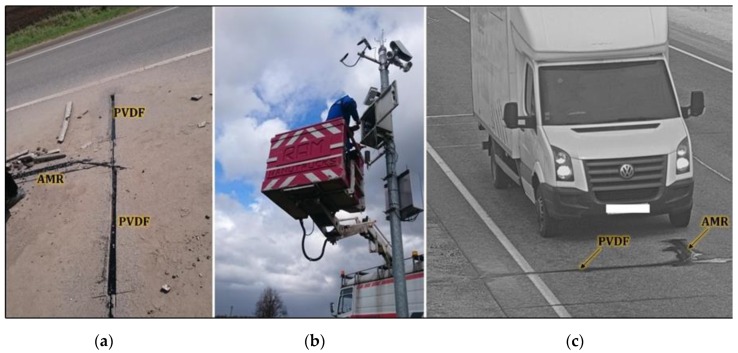
Deployment of traffic monitoring system components on the road (**a**,**b**) and a view from a traffic monitoring camera (**c**).

The traffic monitoring system consists of hardware and software components that are placed in different locations. The magnetic and piezoelectric sensors, their cables and a video camera with infrared illuminator cannot be deployed at a large distance. The magnetic sensors S1–S4 should be placed in the middle of the traffic lane under the surface of the roadway at the beginning of the video camera zone. The general scheme of the traffic monitoring system components deployment is presented in Figure 1 and Figure 2.

### 2.1. Error Due to Discretization in Time

**Figure 3:**
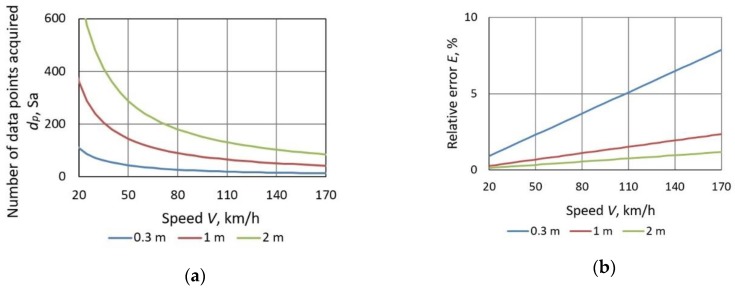
The number of data points acquired in the function of vehicle speed and corresponding error due to discretization (at sampling frequency of 2 kHz): (**a**) the number of data points acquired at different distances between sensor nodes; (**b**) relative error due to discretization at different distances between sensor nodes.

However, there are limitations in the realization of the scheme in Figure 1, i.e., the distance between the magnetic sensors must be optimal. It is determined by several contradictory factors. The larger the distance between the sensors, the more accurately the average speed of vehicle traveling between them can be calculated (Figure 3a). The number of data points acquired during the total time elapsed is defined by the following formula:

(1) $$d_{p}=f_{s}\frac{D}{V},$$

where *D*—distance between two sensor nodes [m], *V*—average speed [m/s], *f*_s_—sampling frequency [Hz].

Sampling frequency should be as high as possible. Errors resulting from the manufacturing process, which cause error of *D*, should be minimum. The relative error *E* due to discretization depends on the sampling frequency, which in this case is 2 kHz (sampling interval 0.5 ms). This error is most significant when vehicles move at higher speeds. It is defined as:

(2) $$E=\frac{1}{d_{p}}100\%.$$

Obviously, increasing the distance between the sensors reduces the sampling discretization error. This error does not exceed 1.2% when sensors are distant by 2 m even at 170 km/h (Figure 3b). However, such a large distance causes a technical problem of situating the sensors at proper places, as their geometric configuration affects the measurement accuracy.

### 2.2. Error Related to Vehicle Deceleration or Acceleration

The location of sensor nodes is an important determinant of the system performance properties [12]. A single-lane configuration and a slight intrusion in the road surface were assumed. The acceptable distance between sensors is from 0.3 m to 1.0 m, because with a greater distance, there is a possibility of additional errors due to a change in vehicle trajectories and accelerations. When the distance between the sensors is approximately 2 m and higher, the speed of a decelerating or accelerating vehicles in the sensor area will be unequal for the first magnetic sensor and the second one. These errors are less significant for vehicles at higher speeds but it should be taken into account for relatively slow moving vehicles. The average rate of acceleration of all the ordinary vehicles is between 1 m/s^2^ and 4 m/s^2^ [13]. Acceleration, considering real models, is varied and depends on a vehicle type and a gear number [14]. Therefore, in previous works [9,10,11], the obtained speed accuracy was determined within the range of ±3 km/h and the comparison to existing systems showed it was sufficient, keeping in mind that the distance between sensors was set to 0.3 m. Due to a low sampling frequency and a short distance between sensors, the accelerating or decelerating effect will not be analyzed in the further sections of this paper.

### 2.3. Trajectory Error

**Figure 4:**
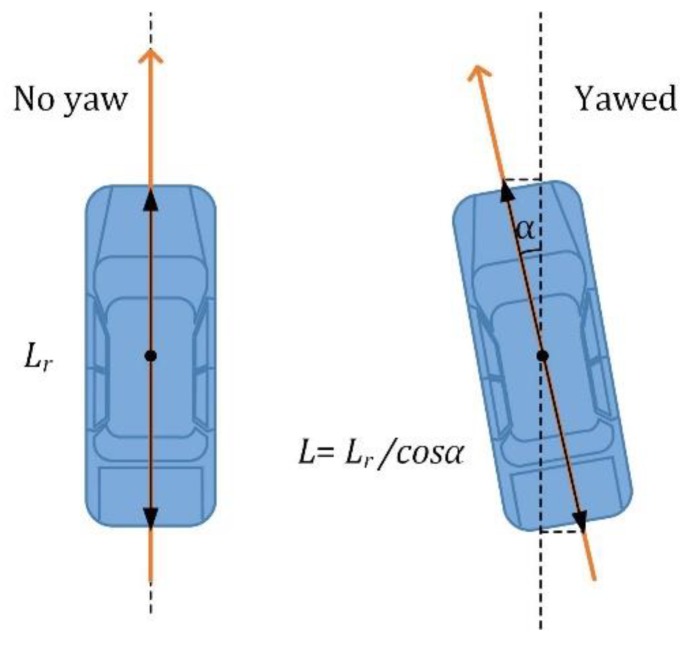
A situation when a vehicle is headed in a different direction than it is pointing.

If the system sensors are deployed at a distance greater than 0.5 m, then there may be an error resulting from vehicle maneuvering (Figure 4). In a situation when a vehicle slip angle α is 30°, the relative error of the estimated length is about 15%. When the cars move on trajectories whose directions do not coincide with the longitudinal axis of the magnetic sensors due to the interaction between the parts of the vehicle and the sensors, the magnetic signatures at the input and at the output of the system may be different. An error occurs when the calculating time delays on the basis of such signatures. The trajectory error may occur with improper sensors installation or due to potholes in a road surface or unknown objects which cause drivers to change direction. They force drivers to turn the steering wheel while entering the sensing area. When installing magnetic sensors in places where turning is prohibited these errors can be reduced (but not eliminated). Magnetic sensors are currently mounted on the longitudinal axis of the lane, but this does not mean that most cars move in the trajectory where the longitudinal axis of the car coincides with the central axis of the lane. In every sensor mounting location a vehicle trajectory survey should be performed to determine the optimum position of the magnetic sensors in relation to the axis of the lane.

### 2.4. Error of Time Delay Estimation

The magnetic flux density *B* (the magnitude of the magnetic field) is the number of magnetic lines of force passing through a unit area of material.

The AMR sensor measures the incident magnetic field *B* in three geometrical axes providing three corresponding components (*B*_x_, *B*_y_, *B*_z_). The changes in the Earth magnetic field magnitude at two sensor nodes, caused by a traveling vehicle, are expressed as follows:

(3) $$\left|{\vec{B}}_{1}\right|=\sqrt{B_{1x}^{2}+B_{1y}^{2}+B_{1z}^{2}}=\;B_{1},\;\left|{\vec{B}}_{2}\right|=\sqrt{B_{2x}^{2}+B_{2y}^{2}+B_{2z}^{2}}=\;B_{2}.$$

For calculating vehicle speed, it is necessary to find the time delay between two curves (data points from two AMR sensors).

The problem gets more complicated in practice as the received signal is corrupted with additive random noise. The result deeply depends on the sample size [10] and preprocessing techniques of signals (i.e., filtering, normalization). Therefore, two curves, *B*_1_ and *B*_2_, can be considered as signal *s* with measurement errors *e_B_*_1_, *e_B_*_2_:

(4) $$B_{1}[n]=s[n]+e_{B1}[n],$$

(5) $$B_{2}[n]=s[n-n_{d}]+e_{B2}[n],$$

where *n*—sample number, *n_d_*—sample delay.

It is commonly accepted, that for this purpose, cross-correlation is one of the most reliable methods [9]. The averaged time delay in seconds is the output of the cross-correlation function. It is given by the following formula:

(6) $$\Delta t={\bar{n}}_{d}\cdot t_{s}=\underset{l}{\arg\max}f[l]\cdot t_{s},$$

(7) $$f[l]=\sum_{n=0}^{N-1}B_{1}[n]\cdot B_{2}[n-l],\begin{array}{cc} & -(N-1)<l<N-1 \end{array},$$

where ${\bar{n}}_{d}$—location of the highest peak of the cross-correlation function *f* (averaged sample delay), *t_s_*—sampling period (1/*f_s_*), *l*—lag (a delay in samples), *N*—total number of samples in the time series.

The averaged sample delay corresponds to the average speed of a vehicle in the time interval between passing sensors S1 and S2 or sensors S1 and S4 (Figure 1). The algorithm for estimating a result variation in the presence of measurement errors is complicated. The analytical error estimation is presented in article [15]. The use of cross-correlation with Fast Fourier Transform (FFT) interpolation, parabolic fit interpolation or the FFT with Hilbert transform can diminish the error of the time delay estimation [16]. Another technique is proposed in the further section of this paper.

## 3. Methods to Estimate Vehicle Speed and Length

### 3.1. Speed Estimation Method

The method is based on calculating derivatives of two scalar functions with respect to time. In the numerical analysis, if the samples are contained in one-dimensional (1D) arrays *B*_1_ and *B*_2_, then, the difference quotients Δ*B*_1_ and Δ*B*_2_ can be estimated using the equation:

(8) $$\Delta B_{1}[n]=\frac{B_{1}[n+\Delta n]-B_{1}[n]}{\Delta n},\;\Delta B_{2}[n]=\frac{B_{2}[n+\Delta n]-B_{2}[n]}{\Delta n},$$

where *n*—subsequent sample number in the computational algorithm, Δ*n*—increment in *n*.

**Figure 5:**
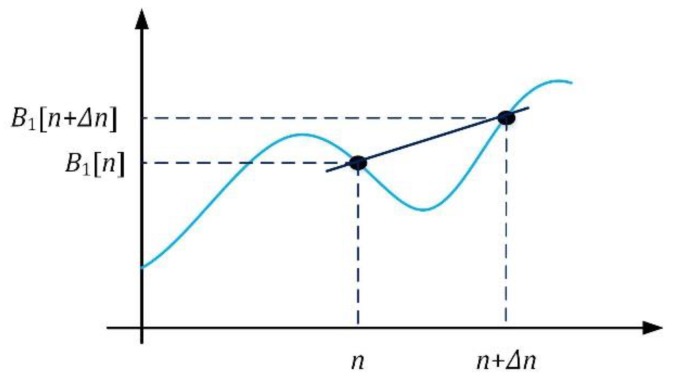
Finding the slope of the secant line of a magnetic field magnitude curve.

As is shown in Figure 5, the difference quotient is used to calculate the slope of the secant line between two points on the graph of functions *B*_1_ and *B*_2_.

**Figure 6:**
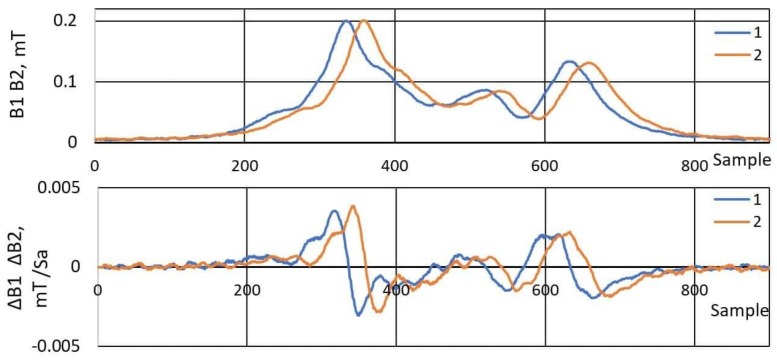
A pair of registered magnetic signatures from sensors S1 and S2 before and after differentiation (Volkswagen Passat 2003, sample delay—23, speed—94 km/h).

As opposed to previously published articles [9,10,11], it was decided to differentiate the change in magnetic field magnitude signals (Figure 6). Then, the cross-correlation function (Figure 5) with inputs Δ*B*_1_ and Δ*B*_2_ instead of *B*_1_ and *B*_2_ was used. Signals *B*_1_ and *B*_2_ were previously affected by signal smoothing with a low-pass filter (100 Hz). Furthermore, the increment Δ*n* plays a significant role in the obtained result of the speed and its accuracy. It will be presented in Section 4.

### 3.2. Length Estimation Method

**Figure 7:**
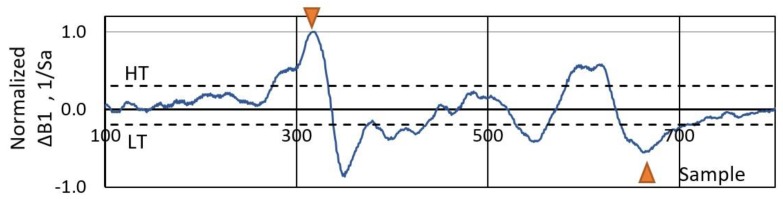
Vehicle length estimation (*L*_1_ = 4.40 m) based on differentiation of the signal from sensor S1. Markers indicate the first and the last significant peaks which represent a vehicle arrival and departure, respectively. HT and LT mean higher and lower threshold, respectively (*f_s_* = 2 kHz).

Estimating a vehicle length is based on two parameters: speed and length of the signal. Since the magnetic signal magnitude rises gradually with an arriving vehicle, it is not a trivial task to accurately identify the arrival and departure moments. Several different methods were tested, and finally, it was chosen to calculate the derivative of a signal from a single sensor. The algorithm relies on the assumption that the first positive significant peak represents the vehicle front and the last negative peak—its rear part. This method is presented in Figure 7. Firstly, the derivative signal is obtained and normalized. Then, the peak finding algorithm is employed. It is crucial to identify the correct peak; therefore, there are two rules: the peak must be above/below a certain threshold value, moreover, the value and the location of the local maximum must be found in a set of data. The second rule enables establishing the first/last peak just above the noise level. Furthermore, since one measurement node consists of two magnetic sensors, it enables the calculation of vehicle length from two independent signals and use those result as the average.

**Figure 8:**
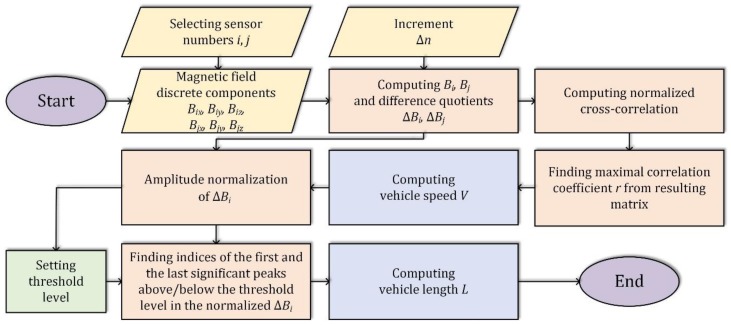
Algorithm for estimating vehicle speed and length.

Figure 8 shows the full signal processing algorithm.

After detecting a vehicle, the threshold HT = −LT was calculated individually for every magnetic signature based on the normalized signal Δ*B_i_*. For this purpose, the highest peak of this signal was found and its value was multiplied by a coefficient. This coefficient represents a percentage. The results in Section 4 are presented for the threshold of 10%.

## 4. Results

### 4.1. Speed Estimation Error

The methods mentioned in Section 3 were verified using a data set of the size of *M* = 290 vehicles. The *MAE* (Mean Absolute Error) is an absolute difference of the reference speed *V_r_* and the measured speed *V_p_*, expressed by the formula:

(9) $$MAE_{p}=\frac{1}{M}\sum_{m=1}^{M}\left|V_{pm}-V_{rm}\right|,$$

where M—number of vehicles which equals 290, m—vehicle number in the data set.

It should be noted that *V_r_* is a value obtained due to the use of PVDF piezoelectric sensors (Roadtrax BL, Measurement Specialties Inc., Hampton, VA, USA) based on the time difference between the front and rear axles. While *V_p_* (for *p* = 1…5) is estimated by means of AMR sensors (LIS3MDL, STMicroelectronics Inc., Santa Clara, CA, USA), which are placed at the distance *D* (Table 1).

The *RMSE* (Root Mean Squared Error) is defined as follows:

(10) $$RMSE_{p}=\sqrt{\frac{1}{M}\sum_{m=1}^{M}{\left(V_{pm}-V_{rm}\right)}^{2}}.$$

**Figure 9:**
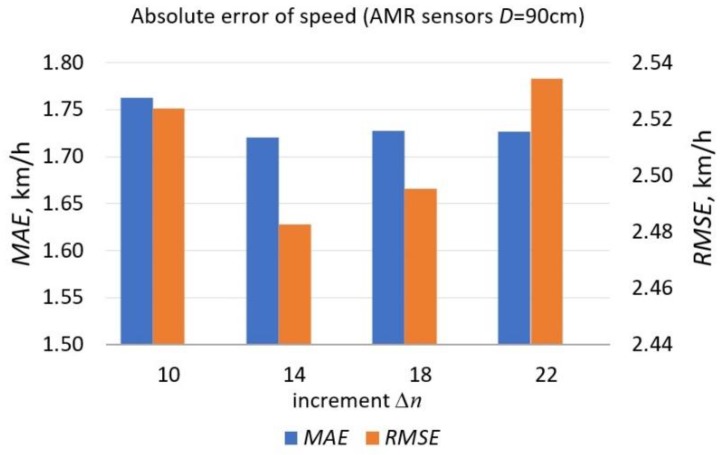
The *MAE* and the *RMSE* of the estimated speed and their variability due to increment Δ*n.* The chart was created for *M* = 290.

Speed estimation is based on cross-correlation which is a measure of similarity of two series as a function of the displacement of one relative to the other. In Figure 9, the *MAE* and the *RMSE* of the estimated speed basing on the series of two AMR sensors distant from each other by *D* = 90 cm, is presented.

As is shown in Table 1, the *MAE* depends on a pair of sensors location in the system as well as the distance between them. The lowest value (*MAE*_5_) is for *D* = 90 cm. The lowest results are obtained when calculations are based on the data from S2 and S3 sensors (*MAE*_3_). The lowest *MAE* occurs for the increment Δ*n* = 14 samples. In Table 2 *MAE* values, which were calculated for three speed ranges (45 km/h–74.99 km/h, 75 km/h–89.99 km/h, 90 km/h–130 km/h), are presented. Speed ranges are based on the road traffic regulations in different countries for dense built-up areas, outside of dense built-up areas, and on motorways. As it can be seen, the lowest *MAE* values are for vehicles at speeds within the range of 45 km/h–74.99 km/h. The *MAE* is strictly dependent on the speed value.

### 4.2. Length Estimation Error

Since the system contains four sensor nodes (Figure 1), a vehicle travel time over each AMR sensor S1–S4 needed to be estimated. For each sensor the number of samples *T*_1_–*T*_4_ in Table 3 represents a period of time between the first maximum and the last minimum of the derivative signal. It is depicted in Figure 7 for sensor S1.

The speed *V_5_* is calculated with the use of the cross-correlation method and the derivative data points from sensors S1 and S4 (*D* = 90 cm). Lengths *L*_1_ and *L*_3_ are calculated according to (11). A vehicle is identified by means of a video camera. *Lr* is a reference length, *W* is a vehicle wheelbase, *G* is a ground clearance—all these parameters are based on the data from vehicle manufacturers. *W*/*Lr* is the wheelbase to length ratio. Lengths *L*_1_ an *L*_3_ are defined in meters as follows:

(11) $$L_{1}=\frac{1000V_{r}T_{1}t_{s}}{3600},\;L_{3}=\frac{1000V_{r}T_{3}t_{s}}{3600}.$$

**Figure 10:**
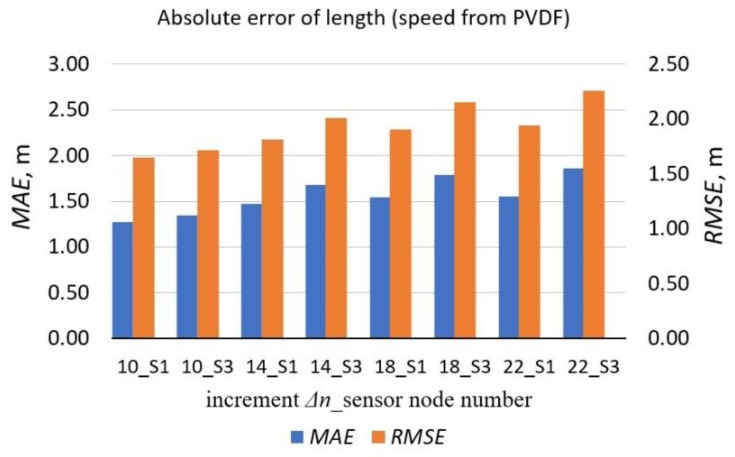
The *MAE* and the *RMSE* of estimated lengths *L*_1_ and *L*_3_ and their dependence on the increment Δ*n* when calculating the difference quotient from the change in magnetic field magnitude data points. Calculations were based on the speed readings from PVDF sensors. The chart was made for *M* = 290.

As presented in Table 3, the differences in samples *T*_1_–*T*_4_ between markers, which indicate the first significant peak and the last one, are quite large. Therefore, *L*_1_ and *L*_3_ are usually not equal. Lengths and their errors were computed using *T*_1_ and *T*_3_ data from sensor nodes S1 and S3. However, it is possible to obtain a result as their average or even the average of *T*_1_-*T*_4_. However, it would lengthen the processing time of a vehicle. This is unwanted, especially for long vehicles travelling at slow speeds. Errors based on S3 are larger than S1 (Figure 10). The *MAE* measures of *L*_1_ and *L*_3_ are defined in the following way:

(12) $$MAE_{L1}=\frac{1}{M}\sum_{m=1}^{M}\left|L_{1m}-L_{rm}\right|,\;MAE_{L3}=\frac{1}{M}\sum_{m=1}^{M}\left|L_{3m}-L_{rm}\right|.$$

When *Vr* speed was used in (11), the lowest value of *MAE* was for the increment Δ*n* = 10 samples but the error differences, in comparison to Δ*n* = 14, 18, 22, were small.

**Figure 11:**
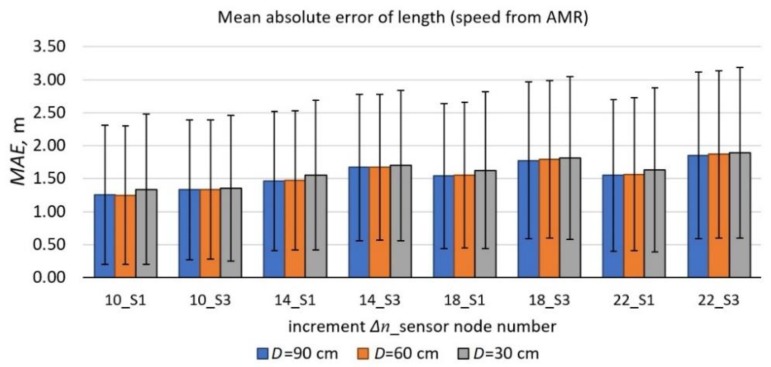
The *MAE* of estimated lengths *L*_1_ and *L*_3_ and their dependence on the increment Δ*n* when calculating a numerical derivative from the change in the magnetic field magnitude data points. Calculations were based on the speed readings from AMR sensors. The chart was made for *M* = 290. The error bars represent the 68% confidence interval of the mean absolute error (*MAE*).

When the speeds from AMR sensors data were used in (11) and the sensors were 60 cm or 90 cm distant from each other, the obtained length errors were up to 8% lower in comparison to the 30 cm distance (Figure 11). The highest *MAE* was about 1.9 m. Finally, the vehicles were divided into five classes according to their length. It is difficult to find one unified vehicle classification in the world. There are commonly used terms of vehicles market segments with or without the number of axles [17]. There are also legal classifications based on interior volume index or gross vehicle weight rating. We decided to calculate the mean *W*/*Lr* ratio for every vehicle class and present it in Table 4. For the longest vehicles, it is noticeably larger.

In Table 5, the *MAE* values are presented for every class and for all classes together. Additionally, for classes A, B, C, the influence of the ground clearance G with W/*Lr* is analyzed and special cases are selected to highlight the differences.

As is shown in Table 6, the adaptive method based on differentiating signals causes a minor decrease of the *MAE* values compared to a fixed threshold-based method [6]. It is especially noticeable for the vehicles with *W*/*Lr* ≥ 0.61. However, for the vehicles with *W*/*Lr* < 0.61, a fixed threshold HT = 0.06 mT the *MAE* values are the lowest.

The data in the tables is presented without calibration correction factors. This means that these error values can be reduced.

### 4.3. Correction Factor of Estimated Length

All results obtained from formula (11) were multiplied by the appropriate correction factor C*_f_* to reduce *MAE*:

(13) $$MAE_{L1}=\frac{1}{M}\sum_{m=1}^{M}\left|C_{f}\cdot L_{1m}-L_{rm}\right|.$$

**Figure 12:**
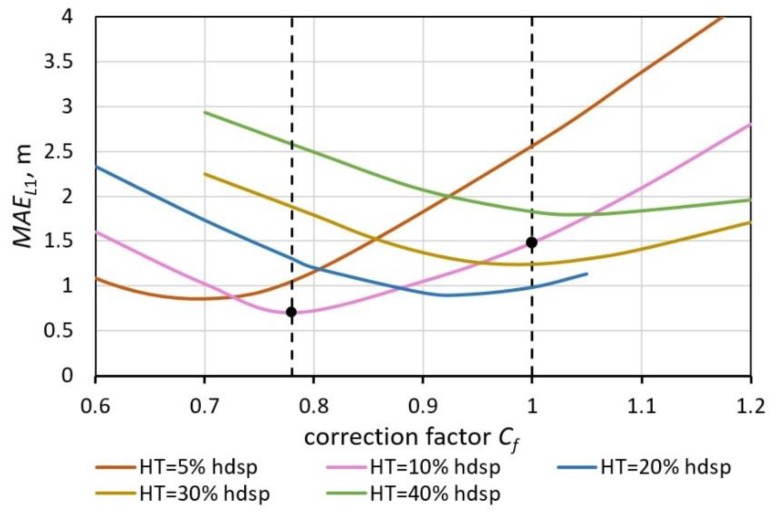
The *MAE* of estimated lengths *L*_1_ and their dependence on the correction factor *C_f_* and threshold HT as a percentage of the highest derivative signal peak. The *MAE* is presented for all classes of vehicles *M* = 290, the signals were smoothed with a 100 Hz low-pass filter.

The dependence of the threshold and the correction factor on the *MAE_L_*_1_ is presented in Figure 12. These parameters need to be selected precisely.

Minimum absolute error values were found for C*_f_* = 0.78 and threshold HT = 0.1 hdsp (hdsp—the highest derivative signal peak). As a result, about 50% error reduction was achieved (Table 7).

### 4.4. Summary

The speed estimation error depends on many factors. One of them is the signal processing technique used. The variability of errors due to the increment Δ*n* when calculating difference quotients is noticeable in Figure 9. Moreover, since the speed is calculated with the use of the cross-correlation function, decreasing the sample size of the processed signals (number of data points) may lead to higher error values. Another factor is the sensor deployment on a road lane. The *MAE* is the lowest (1.7 km/h) for AMR sensors deployed at the largest distance of 90 cm (Table 1). The *MAE* is also the lowest at the speed range of 45 km/h–74.99 km/h (Table 2). It is related to discretization, described in Section 2.1.

The length of a vehicle (11) is estimated based on the time differences between markers (Figure 7) and previously evaluated speed. Every normalized derivative curve contains many peaks, which may be lower or higher. The most important activity which affects accuracy is to setup the adequate threshold. The best results are observed when the calculated length is based on the output data from sensor S1 (Table 3). The errors of the estimated lengths *L*_1_ and *L*_3_ and their dependence on Δ*n* when calculating numerical derivative from the change in the magnetic field magnitude data points are presented in Figure 10 and Figure 11. The *MAE* is the lowest when Δ*n* = 10 samples and *D* = 90 cm.

Finally, the vehicles were divided into 5 classes. The *MAE* of the estimated lengths of all vehicles from a given class and the values of these errors for two wheelbase to the length *W*/*Lr* ratio intervals (lower and upper than the mean for every class) and two ground clearance G intervals are presented in Table 5. These values are from 0.44 m to 2.93 m. This range is from 0.52 m to 1.18 m when using the appropriate calibration factor. The most significant difference is visible between Class C vehicles with *G* ≤ 0.13 m (sedans and wagons) and *G* ≥ 0.18 m (large SUVs). The largest values are for the longest D and E class vehicles in comparison with A, B, C class passenger cars. Differences of *MAE*_L1_ and *MAE*_L3_ for the same *W*/*Lr* and *G* sometimes even reach 40 cm.

The adaptive method based on differentiating signals causes a minor decrease of the *MAE* values compared to a fixed threshold-based method. It should be noted that one of the most important parameters is Δ*n*, which should be 14 samples for our data. The appropriate correction factor *C_f_* is also needed. There is almost no vehicle acceleration or deceleration effect on the *MAE* because the distance between sensors is small (max. 90 cm). On the other hand, there is rather no possibility to enlarge the sampling frequency. There are few digital three-axis sensors, i.e., LIS3MDL, HMC5983, LSM9DS1 and other commercial off-the-shelf products [18] with fast output rate (ODR > 80 Hz) setting.

## 5. Conclusions

Effective traffic management schemes that improve traffic flow and utilization of road infrastructure are very important at present times [19,20]. Magnetic sensors have advantages over other non-contact based sensing techniques because the weather conditions do not affect the efficiency of vehicle detection [21,22]. In the literature, the following features are indicated: the low price of detectors and simplicity of their installation. It is possible to identify the type of vehicle (car, bus, truck) because they differ in the degree of the interference field. Therefore, it seems to be necessary to create an intelligent network of sensors that acquire the changes in the Earth magnetic field magnitude. In some literature only one or two sensors are applied roadway or roadside [23,24]. They usually are used to measure vehicle speed or to detect presence of a traveling vehicle using novel algorithms [25].

The presented system contains four AMR sensors to estimate the length of vehicles. The performed research is a preliminary step before vehicle classification [26]. The piezoelectric sensors (PVDF) were used as the reference system. Based on the data from PVDF sensors, we can know precisely the number of vehicle axles and estimate wheelbases, i.e., the distance between the centers of the front and rear axles. Magnetic sensors generate more information if it is necessary to discern cars of the same class, e.g., between a sedan and an SUV. They both may have the same length and wheelbase, but height of the peaks and their location in their magnetic signatures are usually different. Ultimately, we plan that our system will work with AMR sensors only.

Errors of speed and length estimation presented in Table 1, Table 2, Table 5, Table 6 and Table 7 are mostly the result of the imperfect trajectory of a traveling vehicle over sensors, the different construction and geometry of vehicles, the presence of ferromagnetic loads, which may be carried in trucks with trailers, the discretization of the acquired data and the noise in signals. It is worth pointing out that the visual identification of brand and model of a traveling vehicle is based on a video image and the manufacturer’s data. The type of a vehicle and yawing are taken into account, but the vehicle load can change the length evaluation process.

The *MAE* values of the estimated speed are from 1.3 to 6.3 km/h. They depend on the distance between sensors, speed of vehicle and the filtering signals method used. As a comparison, the errors for variously constructed systems with magnetic sensors placed on the road lane at a distance *D* = 6 m are *RMSE* = 5.1 km/h, *MAPE* = 2.65% (*MAPE* - Mean Absolute Percentage Error) [27], and at a distance of 20 cm *MAPE* = 4.16% or 2.10% [28]. With regards to the system with induction loops, in publication [29], accuracy measures were given for four categories of vehicles: *RMSE*—from 2.5 km/h to 8.6 km/h, *MAPE*—from 1.8% to 9%.

As we can see in Table 5, the lowest *MAE* of the estimated length occurred for C class sedans and wagons (0.44 m). The *MAE* for the longest car is about 8.5% (5.2 m). The vehicle clearance *G* has its influence on the result, e.g., if we compare *MAE* values in columns 5 and 6 of class C. The largest relative *MAE* error is observed for class D, i.e., large VANs and minibuses. That means it equals to 25% with an average length of 7 m to 8 m. When the correction factor is used the relative error is 9.75%. The comparable error values are given in publication [30]; however, in our system the sampling frequency is five times higher, the distance *D* is 10 times shorter and the number of sensor nodes is eight times less.

With regards to designing a reliable transportation system, the AMR sensors are an alternative choice to magneto-impedance sensors [19], other magnetic field sensors and inductive loops. They are dedicated to measure small magnetic fields (a few mT). Due to their small size, low cost and low detection limit (nT), they are suitable for communication systems and mobile applications [2]. They can also be considered suitable to be installed in areas where inductive loops cannot be cut in the road surface [12].

## Figures and Tables

**Table 1:** The reference speed and absolute speed differences for five pairs of sensors depicted in Figure 1. Mean absolute errors for a data set of 290 vehicles of different classes.

Vehicle Number	Reference Speed *Vr*, km/h	*p* = 1	2	3	4	5
		*dV*_P#1 *D* = 30 cm, km/h	*dV*_P#2 *D* = 30 cm, km/h	*dV*_S2-S3 *D* = 30 cm, km/h	*dV*_S1-S3 *D* = 60 cm, km/h	*dV*_S1-S4 *D* = 90 cm, km/h
**1**	87.0	0.5	0.5	0.5	0.5	0.6
2	94.3	3.9	3.9	3.9	0.4	1.0
3	90.7	3.2	0.7	3.2	1.2	0.7
4	63.7	1.7	3.8	1.7	1.7	1.7
5	61.8	0.1	0.1	1.8	0.8	0.5
6	103.4	0.5	4.7	4.7	2.0	1.2
7	82.7	3.7	0.4	3.7	2.0	1.5
8	88.2	1.8	1.8	1.8	1.8	0.5
9	85.7	4.3	2.6	4.3	4.3	0.7
10	88.0	2.1	1.6	2.1	2.1	0.8
…	…	…	…	…	…	…
290	97.2	1.0	1.0	10.8	3.3	1.0
	mean *Vr*, km/h	*MAE*_1_, km/h	*MAE*_2_, km/h	*MAE*_3_, km/h	*MAE*_4_, km/h	*MAE*_5_, km/h
	83.2	2.5	2.2	4.6	2.4	1.7

**Table 2:** Mean absolute errors of vehicles for three speed ranges and for five different pairs of sensors depicted in Figure 1.

Speed Range	45–74.99 km/h	75–89.99 km/h	90–130 km/h
*MAE*_1_, km/h	2.0	2.7	2.6
*MAE*_2_, km/h	1.9	2.3	2.4
*MAE*_3_, km/h	3.2	4.0	6.3
*MAE*_4_, km/h	1.8	2.2	3.1
*MAE*_5_, km/h	1.3	1.6	2.0

**Table 3:** The measured values obtained from four AMR sensors and the geometrical parameters of traveling vehicles.

*T*_1_ Samples	*T*_2_ Samples	*T*_3_ Samples	*T*_4_ Samples	*V_5_* km/h	*L*_1_ m	*L_3_* m	*Vr* km/h	*Lr* m	*W* m	*W/Lr* -	*G* m
585	506	566	599	88.77	7.13	6.89	87.70	4.32	2.690	0.62	0.130
440	489	442	470	98.18	5.76	5.79	94.29	3.86	2.500	0.65	0.150
368	534	491	370	93.91	4.76	6.34	93.04	4.59	2.625	0.57	0.119
356	391	364	434	93.91	4.57	4.67	92.37	4.40	2.678	0.61	0.201
546	572	564	468	90.00	6.68	6.90	88.10	4.93	2.843	0.58	0.165
577	606	556	659	84.16	6.53	6.29	81.49	4.32	2.694	0.62	0.150
399	406	449	430	81.00	4.67	5.25	84.22	4.81	2.857	0.59	0.218
487	608	576	589	85.26	5.68	6.72	83.99	4.64	2.835	0.61	0.150
479	462	477	541	96.72	6.46	6.43	97.03	4.82	2.854	0.59	0.155
417	390	419	458	102.86	5.49	5.51	94.73	4.22	2.578	0.61	0.178

**Table 4:** Vehicle classification according to their reference length *Lr* and determination of the mean parameter *W*/*Lr* value.

class	A	B	C	D	E
	*Lr* ≥ 3.5 and *Lr* < 4.5 m	*Lr* ≥ 4.5 and *Lr* < 4.7 m	*Lr* ≥ 4.7 and *Lr* < 5.2 m	*Lr* ≥ 5.2 and *Lr* < 8.0 m	*Lr* ≥ 8.0 m
max *W*/*Lr*	0.66	0.61	0.61	0.69	0.89
min *W/Lr*	0.57	0.56	0.56	0.54	0.53
mean *W*/*Lr*	0.61	0.59	0.58	0.62	0.71

**Table 5:** The *MAE* of the estimated lengths *L*_1_ and *L*_3_ of all vehicles from a given class and the values of these errors for two wheelbase to the length *W*/*Lr* ratio intervals (lower and upper than the mean) and three ground clearance *G* intervals.

**class A—city cars, small passenger cars, compact SUVs**					
Error (m)	all *W*/*Lr* all *G*	all *W*/*Lr* *G* ≤ 0.13 m	all *W*/*Lr* *G* ≥ 0.18 m	*W*/*Lr* < 0.61 all *G*	*W*/*Lr* < 0.61 *G* ≤ 0.13 m
*MAE_L_*_1_	1.14	1.16	1.37	1.31	1.22
*MAE_L_*_3_	1.49	1.66	1.39	1.62	1.83
**class B—family cars, mid-size SUVs**					
Error (m)	all *W*/*Lr* all *G*	all *W*/*Lr* *G* ≤ 0.13 m	all *W*/*Lr* *G* ≥ 0.18 m	*W*/*Lr* ≥ 0.59 all *G*	*W*/*Lr* ≥ 0.59 *G* ≥ 0.18 m
*MAE_L_*_1_	1.30	1.20	1.74	1.65	1.87
*MAE_L_*_3_	1.58	1.15	2.10	1.52	2.23
**class C—** **executive cars, luxury cars, large SUVs**					
Error (m)	all *W*/*Lr* all *G*	all *W*/*Lr* *G* ≤ 0.13 m	all *W*/*Lr* *G* ≥ 0.18 m	*W*/*Lr* ≥ 0.58 *G* ≤ 0.13 m	*W*/*Lr* ≥ 0.58 *G* ≥ 0.18 m
*MAE_L_*_1_	0.96	0.51	1.95	0.44	1.92
*MAE_L_*_3_	1.05	0.66	2.06	0.49	2.06
	**class D**	**class E**	**all classes of vehicles (A, B, C, D, E)**		
Error (m)	all *W*/*Lr* all *G*	all *W*/*Lr* all *G*	all *W*/*Lr* all *G*	*W*/*L <* 0.61 all *G*	*W*/*Lr* ≥ 0.61 all G
*MAE_L_*_1_	1.95	2.89	1.48	1.21	2.06
*MAE_L_*_3_	2.16	2.93	1.68	1.40	2.27

Class B, column 6—ANs: Volkswagen Sharan, Ford Galaxy, etc.; class C, column 5—sedans and wagons: BMW 5, Mercedes-Benz E cl., VW Passat, etc.; class C, column 6—large SUVs: Volvo XC90, Porsche Cayenne, Mercedes-Benz M cl., etc.; class D—large VANs, minibuses; class E—three or more axle vehicles, buses.

**Table 6:** The *MAE* of estimated lengths *L*_1_ and *L*_3_ of all vehicles—comparison of two methods.

all classes of vehicles (A, B, C, D, E)			
Error (m)	all *W*/*Lr* all *G*	*W*/*L <* 0.61 all *G*	*W*/*Lr* ≥ 0.61 all *G*
fixed threshold-based method (signals not differentiated, HT = 0.03 mT)			
*MAE_L_*_1_	1.76	1.54	2.17
*MAE_L_*_3_	1.75	1.55	2.13
fixed threshold-based method (signals not differentiated, HT = 0.06 mT)			
*MAE_L_*_1_	1.43	1.00	2.44
*MAE_L_*_3_	1.42	0.95	2.57
adaptive two-extreme-peak detection method (signals differentiated, Δ*n* = 14, HT = 0.1 hdsp)			
*MAE_L_*_1_	1.48	1.21	2.06
*MAE_L_*_3_	1.68	1.40	2.27

**Table 7:** The *MAE* of estimated lengths *L*_1_ and *L*_3_ of all vehicles after multiplying by C*_f_* = 0.78.

all classes of vehicles (A, B, C, D, E)			
Error (m)	all *W*/*Lr* all *G*	*W*/*L <* 0.61 all *G*	*W*/*Lr* ≥ 0.61 all *G*
adaptive two-extreme-peak detection method (signals differentiated, Δ*n* = 14, HT = 0.1 hdsp)			
*MAE_L_*_1_	0.70	0.64	0.80
*MAE_L_*_3_	0.75	0.67	0.92
